# Profilings of MicroRNAs in the Liver of Common Carp (*Cyprinus carpio*) Infected with *Flavobacterium columnare*

**DOI:** 10.3390/ijms17040566

**Published:** 2016-04-15

**Authors:** Lijuan Zhao, Hong Lu, Qinglei Meng, Jinfu Wang, Weimin Wang, Ling Yang, Li Lin

**Affiliations:** 1 Shandong Freshwater Fisheries Research Institute, Jinan 250013, China; zhaolijuan4234@163.com (L.Z.); luhong.518@163.com (H.L.); qingleimeng@126.com (Q.M.); wangjinfu1978@163.com (J.W.); 2 Shandong Provincial Key Laboratory of Freshwater Genetics and Breeding, Jinan 250013, China; 3 Shandong Provincial Freshwater Aquatic Products Quality Inspection Center, Jinan 250013, China; 4 Key Lab of Freshwater Animal Breeding, Ministry of Agriculture, Huazhong Agricultural University, Wuhan 430070, China; wangwm@mail.hzau.edu.cn; 5 Department of Aquatic Animal Medicine, College of Fisheries, Huazhong Agricultural University, Wuhan 430070, China

**Keywords:** *Flavobacterium columnare*, common carp, liver, miRNA, deep sequencing

## Abstract

MicroRNAs (miRNAs) play important roles in regulation of many biological processes in eukaryotes, including pathogen infection and host interactions. *Flavobacterium columnare* (FC) infection can cause great economic loss of common carp (*Cyprinus carpio*) which is one of the most important cultured fish in the world. However, miRNAs in response to FC infection in common carp has not been characterized. To identify specific miRNAs involved in common carp infected with FC, we performed microRNA sequencing using livers of common carp infected with and without FC. A total of 698 miRNAs were identified, including 142 which were identified and deposited in the miRbase database (Available online: http://www.mirbase.org/) and 556 had only predicted miRNAs. Among the deposited miRNAs, eight miRNAs were first identified in common carp. Thirty of the 698 miRNAs were differentially expressed miRNAs (DIE-miRNAs) between the FC infected and control samples. From the DIE-miRNAs, seven were selected randomly and their expression profiles were confirmed to be consistent with the microRNA sequencing results using RT-PCR and qRT-PCR. In addition, a total of 27,363 target genes of the 30 DIE-miRNAs were predicted. The target genes were enriched in five Kyoto Encyclopedia of Genes and Genomes (KEGG) pathways, including focal adhesion, extracellular matrix (ECM)-receptor interaction, erythroblastic leukemia viral oncogene homolog (ErbB) signaling pathway, regulation of actin cytoskeleton, and adherent junction. The miRNA expression profile of the liver of common carp infected with FC will pave the way for the development of effective strategies to fight against FC infection.

## 1. Introduction

*Flavobacterium columnare* (FC) is a member of the family Flavobacteriaceae and was identified as the causative agent of columnaris. It can cause world-wide fish diseases with high mortality and heavy economical losses in aquaculture industry, including channel catfish (*Ictalurus punctatus*), grass carp (*Ctenopharygodon idella*), Mandarin fish (*Siniperca chuatsi*), and common carp (*Cyprinus carpio*), *etc.* [1,2]. Common carp is the number one fish of aquaculture in the world with the annual products amounting to approximately three million tonnes [3]. In recent years, an increased incidence of common carp columnaris was reported [4]. Identification of host proteins and miRNAs which are involved in response to FC infection has a great significance for the prevention of columnaris in common carp. MiRNAs are small RNAs with approximately 22 nucleotides in length. MiRNA are excised from RNA precursors (pre-miRNAs) by t enzyme Dicer [5]. The pre-miRNAs are processed from the primary RNA (pri-miRNAs) in the nucleus by enzyme Drosha [6]. One of the duplex strands of the mature miRNAs can be incorporated into the RNA-induced silencing complexes (RISCs), thereafter, they bind to the 3^′^ untranslated region (3^′^UTR) of the target mRNAs, so as to degrade the target mRNAs or inhibit their translation [7]. It has been well established that miRNAs are involved in regulation of many biological processes in eukaryotes, including pathogen infection and host interactions [8]. Concerning the miRNAs of common carp, there have been three reports [9,10,11]. In these previous reports, the authors identified miRNAs from the muscle, spleen, and mixed tissues of common carp without any pathogen infection, but no study on miRNAs from the liver (a major target of FC infection) of common carp has been reported. Furthermore, the complete genome of common carp was not published when they analyzed the miRNAs of common carp in the three previous reports. Systematic analysis on the miRNA of common carp involved in FC infection is not available. Ultrahigh-throughput sequencing is a powerful tool to explore genome-wide transcriptomic analysis at high resolution. The miRNAs profiles of many fish have been characterized by this technique [12]. In the present report, we present the miRNA profiling of the liver from common carp infected with or without FC. Additionally, a great number of miRNAs target genes were annotated and enriched pathways were analyzed. The target genes were enriched in focal adhesion, ECM-receptor interaction, ErbB signaling pathway, regulation of actin cytoskeleton, and adherent junction KEGG pathways which are closely related to the bacterial infections [13]. These results shed a new light on the development of effective strategies to fight against FC infection.

## 2. Results

### 2.1. Construction of cDNA Library for Sequencing and Small RNA Discovery

**Figure 1:**
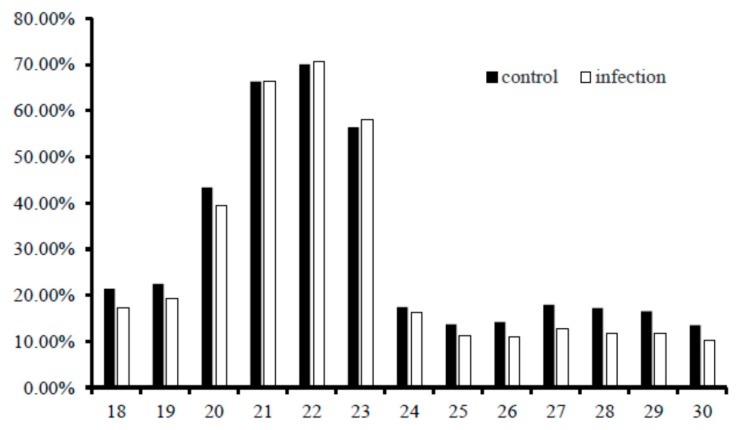
Length distribution of miRNA mapped rates in livers of common carp infected with *Flavobacterium columnare* (FC) and control. The miRNA mapped rates from the two samples with length between 18 and 30 nt were analyzed. The 100% was the clean read count of small RNAs of a certain length.

To investigate the miRNA expression profiles of the liver of common carp, the cDNA library of small RNAs was constructed with pooled total RNAs from liver tissues collected from 10 common carps infected with FC or control. Through IlluminaHiseq2500, 19,619,555 and 18,776,497 raw reads were obtained from control and FC-infected samples, respectively; After removal of low quality reads (the 5^′^ and 3^′^ adapters, reads containing ploy-N, reads with sequences smaller than 18 nt or longer than 30 nt), 16,907,877 (94.94%) and 13,599,310 (94.58%) high-quality (Q30) clean reads were extracted from control and infected samples, respectively (Table 1). Using Bowtie software searches against Silva database, GtRNAdb database, Rfam database, and Repbase database, 3,210,400 (18.99%) and 3,758,445 (27.64%) of the clean reads from control and infected samples, respectively, were annotated as either repeat base, rRNA, snRNA, snoRNA, scRNA or tRNA. Given the genome of common carp is available, the clean reads small RNAs 16,907,877 (control) and 13,599,310 (infected) were mapped to the common carp genome using miRDeep2 software. In the case of the control sample, 9,181,153 (54.3%) of miRNA clean reads were mapped to the common carp genome. While in the infected samples, 6,479,172 (47.6%) of miRNA clean reads were mapped to the common carp genome (Table 1). The length distribution of the miRNA mapped rate was analyzed and a similar trend of the distribution was observed in the two samples. Higher miRNA mapped rates were observed in small RNAs with 21–23 nt in length (Figure 1).

### 2.2. Identification of miRNAs and Validation by RT-PCR and qRT-PCR

**Figure 2:**
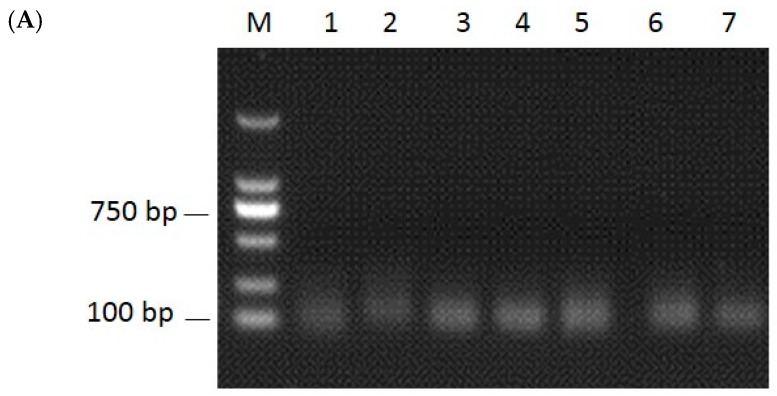

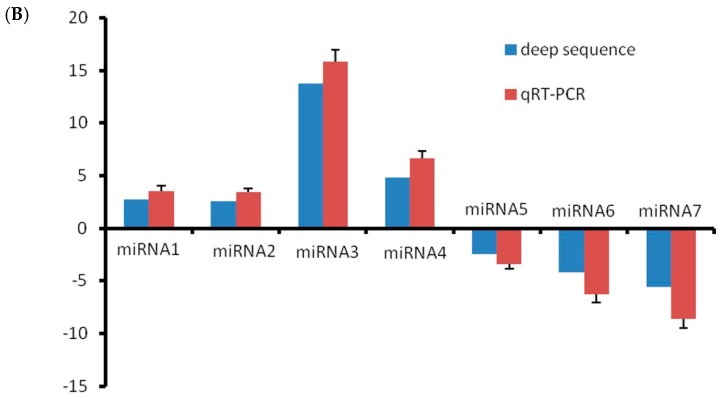
Validation of seven novel miRNAs by RT-PCR and qRT-PCR. M: marker; lane 1–7: the RT-PCR products of the seven novel miRNAs. (**A**) RT-PCR results; (**B**) qRT-PCR results.

To identify miRNAs in the liver of the common carp infected with or without FC, the clean reads were used and the miRNAs identified by comparing with the deposited miRNAs from miRBase using Bowtie tools software. Randfold tools soft was used for novel miRNA secondary structure prediction. There were totally 698 miRNAs, including 142 deposited miRNAs and 556 predicted novel miRNAs (Table S1). Among the deposited miRNAs, 13 miRNAs (Ccr-miR-22a, ccr-miR-122, ccr-miR-146a, ccr-miR-192, ccr-miR-148, ccr-miR-100, ccr-miR-21, ccr-miR-126-3p, ccr-miR-143, ccr-miR-26a, ccr-let-7a, ccr-miR-101a, and ccr-miR-199-5p) were the most abundant miRNAs (TPM > 10,000) in both samples (TPM = Readout × 1,000,000/Mapped reads, Table S2). In order to validate the novel miRNAs, RT-PCR was used to test randomly selected seven miRNAs from the control, and the size of RT-PCR products were observed around 100 bp (Figure 2A), indicating the existence of these miRNAs in the liver of common carp.

### 2.3. Conservation Analysis of the Identified Common Carp miRNAs

**Figure 3:**
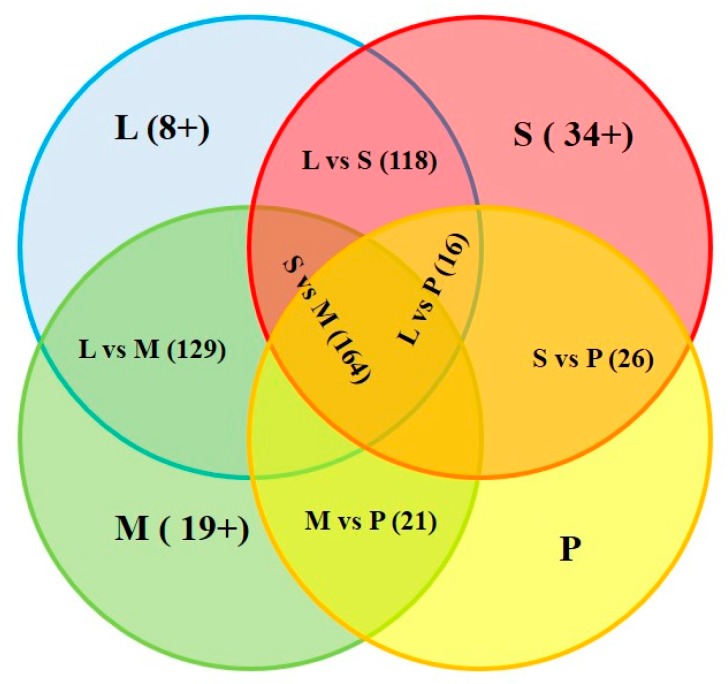
Comparison of miRNAs from different tissues of common carp. The number of over-lapping is shown in the parentheses. Tissue specific miRNA is shown in the parentheses and marked with “+”. L, S, M, and P represents liver, spleen, skeletal muscle and pooled tissues, respectively.

To date, thousands of miRNAs have been annotated in nearly 100 species [14]. In this report, we identified 142 deposited miRNAs and 556 predicted novel miRNAs of common carp. The comparison was further performed between the deposited miRNAs discovered in the common carp with those identified in the previous reports [9,10,11]. The result shows that the number of over-lapped miRNAs between liver and spleen was 118, between liver and skeletal muscle 129, between liver and pooled tissues 16, between spleen and skeletal muscle 164, between spleen and pooled tissues 26, and between skeletal muscle and pooled tissues 21 (Figure 3 and Table 2). Tissue specific miRNA was shown in bold in the Table S1. The number of specific miRNAs detected in the liver, spleen and skeletal muscle was 8, 34, and 19, respectively. One hundred and thirty four miRNAs were discovered in common carp published previously, eight miRNAs were newly identified in the common carp (Table S1), named ccr-miR-124a, ccr-miR-124b, ccr-miR-132b, ccr-miR-7132, ccr-miR-7133, ccr-miR-722, ccr-miR-9-3p, and ccr-miR-9-5p. Considering there were still 556 predicted miRNAs candidates, this indicates that there are still many new miRNAs of common carp remaining to be characterized. To analyze the conservation of common carp miRNA families, we compared them to the other eight animal miRNAs obtained the data from miRbase database (Available online: http://www.mirbase.org/), including worm (*Caenorhabditis elegans*), fly (*Drosophila melanogaster*), zebrafish (*Danio rerio*), channel catfish (*Ictalurus punctatus*), frog (*Xenopus tropicalis*), mouse (*Mus musculus*), chicken (*Gallus gallus*), and human (*Homo sapiens*). As shown in Table 3 and Table S2, miR-let_7, miR-8, miR-1, miR-34, miR-9 and miR-124 families were highly conserved from worm to human, miR-2 was found only in the two invertebrate species (worm and fly), miR-63 and miR-392 were only found in worm. Eight miRNAs were highly conserved from fly to human (No. 10–17), among them, 62.5% (five of eight, miR-10, miR-25, miR-7, miR-375, and miR-29) were abundant ones (>1000 reads). Forty one miRNA species were identified in the nine represented vertebrates (No. 26–66), among them 58.5% (24 of 41) miRNAs were highly expressed (>1000 read, No. 26–49). There were 10 miRNAs which were only identified in common carp (No. 92–101), among them 50% (five of 10, miR-122, miR-722, miR-217, miR-363 and miR-724) were abundant ones (>1000 reads).

### 2.4. Differentially Expressed miRNAs (DIE-miRNAs) between Infected and Control Livers of Common Carp

In the infected samples, 30 miRNAs were differentially expressed including 14 up-regulated and 16 down-regulated ones (Table 4). Among them, 21 were predicted novel miRNAs, while nine miRNAs (miR-196b, miR-365, miR-184, miR-153b, miR-301a, miR-133a-5p, miR-132b, miR-124a, miR-124b) were deposited in the miRbase database (Available online: http://www.mirbase.org/). Based on the similarity of miRNA seed sequences, miRNAs can be classified into many families. Since miRNAs in the same family have similar functions, we further analyzed the expression profiles of the nine deposited DIE-miRNA species at the family level. Only miR-365, miR-184, and miR-124 showed significant expression at both species and family levels. The other six miRNAs only showed significant expression at species level but not at family level (Table 5). For example, miR-301a was significantly up-regulated (3.58 times) in the infected sample. miR-301a belongs to the miR-130 family which consists of four members (miR-301a, miR-130b, miR-130c, miR-130a). When we calculated the expression of all members in the miR-130 family, there was no significantly up-regulated in the infected sample (0.90 times, even a little lower expression). In order to validate the results of the differentially expressed miRNAs, the expression levels of seven randomly selected miRNAs were quantified by qRT-PCR in both samples. The expression profiles of the seven miRNAs were consistent with those obtained by deep sequencing (Figure 2B).

### 2.5. miRNA Target Genes, Annotation and KEGG Pathway Enrichment Analysis

**Figure 4:**
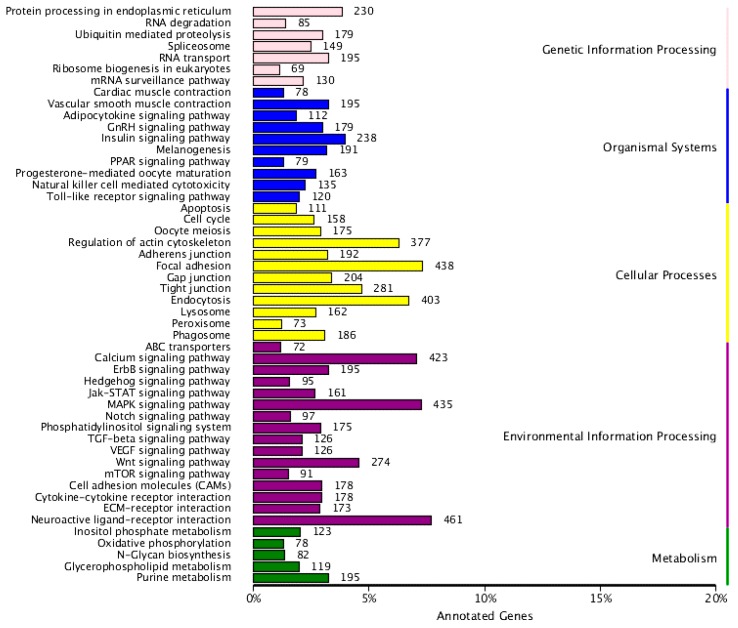
Functional enrichment of differentially expressed miRNA target genes.

**Figure 5:**
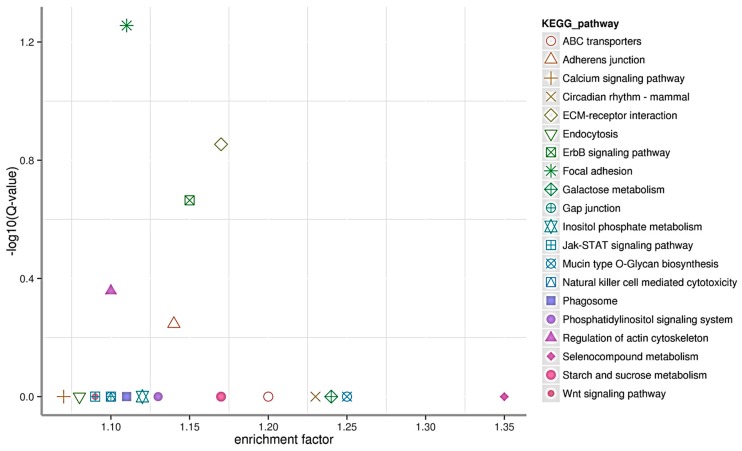
KEGG pathway enrichment of differentially expressed miRNA target genes. KOBAS software (Availible online: http://kobas.cbi.pku.edu.cn/help.do) was used to test the statistical enrichment of differential expression genes in KEGG pathways.

To better understand the functions of miRNAs in the liver of common carp, the target genes of miRNAs in common carp were predicted using miRnada (Available online: http://www.microrna.org) and RNAhybrid (Available online: http://bibiserv.techfak.uni-bielefeld.de/rnahybrid/) softwares. There were totally 52,537 target genes of the identified 698 miRNAs. More than 91.3% (47,982 out of 52,537) of the target genes could be annotated. The 30 DIE-miRNAs target genes were 27,363 which were grouped into genetic information processing, organismal systems, cellular processes, environmental information processing, and metabolism clusters. Furthermore, neuroactive ligand-receptor interaction, focal adhesion, mitogen-activated protein kinase (MAPK) signaling pathway, regulation of actin cytoskeleton, and endocytosis were the top five clusters (Figure 4). When the target genes were further subjected to KEGG pathway enrichment analysis, the results showed that there were five significant enriched pathways as following: Focal adhesion (*Q* = 0.056), ECM-receptor interaction (*Q* = 0.140), ErbB signaling pathway (*Q* = 0.217), Regulation of actin cytoskeleton (*Q* = 0.439), and Adherent junction (*Q* = 0.567) (Figure 5).

## 3. Discussion

MicroRNAs are involved in diverse biogenesis pathways and versatile regulatory functions. They have convoluted relationships, in which they cooperate, compete, or regulate each other [15]. They are involved in basic cellular processes, including differentiation, proliferation, and apoptosis [16]. Common carp is an important commercial fish worldwide. Recently, the whole genome of common carp was sequenced [2]. There have been three reports about the miRNAs of common carp [9,10,11]. However, these miRNAs were identified in common carp without infection and they were published before the genome of common carp was available. *F. columnare* (FC) infection can cause pathogenesis of the liver of common carp with high mortality. However, miRNA profiling of common carp in response to FC infection has not been characterized. In this study, 142 deposited miRNAs and 556 predicted miRNAs were identified from the liver of common carp. Among the 142 deposited miRNAs, eight were first identified in common carp, 134 were identical to those reported previously [9,10,11]. When we compared the miRNAs from nine animals, more than 50% of the conserved miRNAs were highly expressed in common carp (>1000 reads), indicating that these miRNAs might be important in the common carp.

Numerous reports have shown that the expression patterns of miRNA are species, tissue, and time dependent. For example, miR-1and miR-let_7 were expressed in the mature fly but not in the embryo of the fly [17]. Moreover, miR-171 was highly expressed in the flower but not in the leaves of *Arabidopsis thaliana* [18]. In this report, we compared the tissue specificity of miRNAs in common carp. As shown in Figure 3 and Table 2, they were indeed many miRNAs which specifically expressed in the liver, spleen, and skeletal muscle. In the previous report [10], the authors analyzed the miRNAs from pooled 10 tissues which included the three above mentioned tissues. Therefore, it is reasonable to believe that miRNAs in the pooled tissues should cover all miRNAs identified in the liver, spleen, and skeletal muscle. However, for an unknown reason, there were only 80 miRNAs identified in the pooled tissues and most of the miRNAs in the three tissues could not be found.

The DIE-miRNA target genes were grouped into five significant enriched KEGG pathways, named focal adhesion, ECM-receptor interaction, ErbB signaling pathway, adherent junction, and regulation of actin cytoskeleton. Focal adhesions play essential roles in important biological processes including cellular motility. In the focal adhesions, bundles of actin filaments are anchored to the receptors of the integrin signaling pathway, resulting in reorganization of the cytoskeleton which is a prerequisite for changes in cell shape and motility [19,20]. ECM consists of many macromolecules and plays an important role in the maintenance of cell and tissue structure and function. Integrins are involved in the interactions between cells and ECM, and play important roles in the control of cell adhesion, migration, and apoptosis. The ErbB protein family consists of four tyrosine kinases. ErbB signaling is closely related to the neurodegenerative diseases and tumors of humans [21]. Adherent junctions are protein complexes which link to the intracellular actin cytoskeleton. Via direct interaction, adherent junctions play a role as a bridge connecting the cytoskeleton of neighboring cells [22]. Apparently, the above mentioned enriched pathways were all closely related to cytoskeleton and its associated signaling pathways, such as the integrin signaling pathway and the cadherins signaling pathway, indicating that FC infection has dramatic effects on the cell adhesion, migration, differentiation, proliferation, and apoptosis of the liver cells of common carp. The underlying mechanism is not known and remains to be elucidated.

Among the deposited nine DIE-miRNAs, the read counts of three miRNAs (miR-196b, miR-365, miR-184) in both infected and control samples were more than 20 (TPM > 3.5), the read counts of the other six miRNAs in both infected and control samples were less than 20 (TPM < 3.5). Considering the high sensitivity of Highseq 2500 assay, we have only focused our discussion on three miRNAs with read counts more than 20. By comparison with the control sample, ccr-miR-196b and ccr-miR-365 were significantly up-regulated, while ccr-miR-184 was significantly decreased. The functions of the three miRNAs have been intensively studied in the human being. These three miRNAs can suppress or promote the pathogenesis of cancers of humans. miR-184 may have oncogenic effects in liver cancer, glioma cancer, and bone cancer [23,24,25], but has anti-cancer effects on lung cancer, renal, and corneal tumors [26,27,28]. miR-196 had oncogenic functions in colorectal cancer, pancreatic cancer, breast cancer, leukaemia, and oesophageal adenocarcinoma [29,30,31,32,33,34,35,36,37], but acts as a tumor suppressor of melanoma [38]. miR-365 could promote the pathogenesis of squamous cell carcinoma and gastric tumor [39,40], but could inhibit the development of lung cancer, colon cancer, and melanoma [41,42,43].The mechanisms of action of these three miRNAs in different types of cancer are not known. In fish, it has been shown that miR-184 was expressed in the eye of zebrafish embryo [44] and was expressed specifically in retina cells, indicating cell type- and developmental stage-specific expression patterns of miR-184 [45]. miR-196b could target estrogen receptor transcript [46] and estrogens are important in the teleost spermatogonial process [47]. The function of miR-365 in fish is largely unknown. These regulation mechanisms of these three miRNAs involved in FC infection in common carp are enigmatic and warrant further investigation.

## 4. Materials and Methods

### 4.1. Fish, Bacteria, and Infection

*Flavobacterium columnare* (FC) was isolated from diseased yellow catfish (*Pelteobagrus fulvidraco*) and kept in the College of Fisheries, Huazhong Agricultural University. All animal experiments were performed using wild common carps bred in the Shandong Freshwater Fisheries Research Institute in accordance with the recommendations in the Guide for the Care and Use of Laboratory Animals of China and Shandong Freshwater Fisheries Research Institute. Fish were maintained at 25–26 °C in a recirculating freshwater system and acclimatized in the laboratory for 2 weeks before experiments. Fish were divided into two groups, each group consisted of 10 fish. Fish were intraperitoneally infected with FC at the dose of 10^4^ pfu/g.body weight or the same volume of culture medium of FC, and were used as the infected group or control group. Meanwhile, fish liver tissues were collected at 40 h post infection. The samples were snap-frozen in liquid nitrogen and stored at −80 °C.

### 4.2. Small RNA Isolation and cDNA Library Construction

Liver tissues used for the generation of the small RNA library were obtained from10 individual fish from FC-infected and control groups, respectively. Total RNA was isolated from livers using TRIzol reagent (Invitrogen, Carlsbad, CA, USA). The purity, concentration, and integrity of RNA samples were measured to ensure that the RNA quality met the criteria for sequencing using the NanoPhotometer spectrophotometer (IMPLEN, Westlake Village, CA, USA), Qubit 2.0 Flurometer (Life Technologies, Carlsbad, CA, USA) and the Agilent Bioanalyzer 2100 system (Agilent Technologies, Santa Clara, CA, USA), respectively. Equal amount of the RNAs from 10 fish were mixed together to eliminate the variation among the samples and used as a sample. A total amount of 1.5 μg RNA per sample was used as input material for the RNA sample preparations. Sequencing libraries were generated using NEBNext Ultra small RNA Sample Library Prep Kit for Illumina (NEB, Boston, MA, USA). Briefly, mixed 3′ SR Adaptor, RNA and nuclease-free water in a tube and incubated for 2 min at 70 °C in a preheated thermal cycler, followed by transferring the tube to ice. Then, 3′-ligation reaction buffer (2×) and 3′-ligation enzyme mix were added and incubated for 1 h at 25 °C in a thermal cycler. To prevent adaptor-dimer formation, the SR RT Primer hybridized to the excess of 3′ SR Adaptor and transformed the single stranded DNA adaptor into a double-stranded DNA molecule. Then, the first chain of cDNA was synthesized by reverse transcription. Subsequently, the cDNAs were amplified using PCR. The small-RNAs were separated using polyacrylamide gel and purified using the AMPure XP system (Beckman Coulter, Brea, CA, USA). The clustering of the index-coded samples was performed on a cBot Cluster Generation System using TruSeq PE Cluster Kit v4-cBot-HS (Illumina). After cluster generation, the library preparations were sequenced on an Illumina Hiseq 2500 platform (Illumina, Santa Clara, CA, USA) and paired-end reads were generated at Biomaker Biotechnologies, Co., Ltd. (Beijing, China).

### 4.3. Sequence Analysis and Identification of miRNAs

The raw reads were firstly processed through in-house perl scripts. By removing reads containing adapter, ploy-N, and low quality from raw data, the clean reads were obtained. Subsequently, the clean reads were further processed by removing the sequences smaller than 18 nt or longer than 30 nt. Meanwhile, Q20, Q30, GC-content and sequence duplication level of the clean data were measured. Clean data with high quality were used for the downstream analyses.

The clean reads were aligned with Silva, GtRNAdb, Rfam and Repbase database using Bowtie tools software (Available online: http://bowtie-bio.sourceforge.net/) respectively. After filtering ribosomal RNA (rRNA), transfer RNA (tRNA), small nuclear RNA (snRNA), small nucleolar RNA (snoRNA), repeat sequences, and other non-coding RNA (ncRNA), the remaining reads were used to detect known miRNA and new miRNA predicted by mapping to the common carp genome using SOAP (Available online: http://soap.genomics.org.cn). The miRNA precursors were predicted by homologous comparison miRNA sequences to the genome sequence of the common carp with mfold software (Available online: http://unafold.rna.albany.edu). New miRNA secondary structure was predicted using Randfold tools software (Available online: http://www.aquafold.com).

### 4.4. Target Gene Functional Annotation

Gene function was annotated based on the Nr (NCBI non-redundant protein sequences), Nt (NCBI non-redundant nucleotide sequences), Pfam (Protein family).

KOG/COG (Clusters of Orthologous Groups of proteins), Swiss-Prot (manually annotated and reviewed protein sequences), KO (KEGG Ortholog), and GO (Gene Ontology) databases.

### 4.5. Differential miRNA Expression Analysis

Differential expression analysis of miRNAs was performed using the IDEG6 software (Available online: http://telethon.bio.unipd.it/bioinfo/IDEG6_form). *p*-value was adjusted using *q*-value. *q*-value < 0.005 and |log2 (fold-change)| ≥ 1 was set as the threshold for significantly differential expression.

### 4.6. GO and KEGG Pathway Enrichment Analysis

GO enrichment analysis of the differentially expressed genes was implemented by the GOseq R packages (Available online: http://www.bioconductor.org) based Wallenius non-central hyper-geometric distribution. The statistical enrichment of differential expression genes in KEGG pathways was measured using KOBAS software (Available online: http://kobas.cbi.pku.edu.cn). Differential expression analysis of two samples was checked using the IDEG6. *p* value was adjusted using *q* value. *q* value < 0.005 and |log2 (fold change)| ≥ 1 was set as the threshold for significantly differential expression.

### 4.7. RT-PCR and qRT-PCR Analysis of miRNAs

The predicted novel miRNAs were validated by RT-PCR as described previously [48]. The primers used for this study was shown in Table S5. Seven randomly selected miRNAs were detected by qRT-PCR using the same RNA samples used for the construction of the miRNA library. Seven forward primers were designed based on mature miRNA sequences. The reverse primer was supplied in the kit purchased from Tiagen, Beijing. *U6 RNA* gene was amplified as an endogenous control to normalize template amounts. Quantitative PCR reactions were conducted in 20 µL volumes containing 1 µL diluted cDNA, 300 nM of each primer, and 10 µL of the SYBR Master Mix with the following cycling conditions: 95 °C for 5 min, 45 cycles at 95 °C for 10 s, 58 °C for 10 s, and 72 °C for 15 s, and ended with 95 °C at 5 °C/s calefactive velocity to make the melt curve. All expression levels were normalized to the *U6 RNA* gene. Amplification results were analyzed using a comparative *C*_t_ method. *C*_t_ represents the threshold cycle.

## Figures and Tables

**Table 1:** Overview of miRNA high-sequencing data. Mapped rate (%) = mapped miRNA reads/clean reads × 100.

Sample	Clean Reads	Annotated Small RNA	Unannotated Small RNA	Mapped siRNA Reads	Mapped Rate (%)
Control	16,907,877	3,210,400	13,697,477	9,181,153	54.3
Infection	13,599,310	3,758,445	9,840,865	6,479,172	47.6

**Table 2:** Comparison of miRNAs from different tissues of common carp. The number of over-lapped miRNA is followed by the number of tissue specific miRNAs which are in parentheses. L, S, M, and P represent liver, spleen, skeletal muscle, and pooled tissues, respectively.

Tissue	Liver (142)	Spleen (193)	Skeletal Muscle (188)	Pooled Tissues (80)
liver	–	118 (L: 24; S: 75)	129 (L: 13; M: 59)	16 (L: 126; P: 64)
spleen	–	–	164 (S: 29; M: 24)	26 (S: 167; P: 54)
skeletal muscle	–	–	–	21 (M: 167; P: 59)
pooled tissues	–	–	–	–

**Table 3:** Comparison of miRNAs among invertebrates and vertebrates. The miRNAs from other represented animals were obtained from miRbase database (Available online: http://www.mirbase.org/).

No.	miRNA Family	cel	dme	ccr	dre	ipu	xtr	gga	mmu	hsa	ccr Read
**1**	**miR-let_7**	**1**	**1**	**5**	**18**	**21**	**9**	**11**	**12**	**12**	**185,356**
**2**	**miR-8**	**1**	**1**	**3**	**6**	**6**	**3**	**3**	**5**	**5**	**41,287**
3	miR-1	1	1	2	4	3	4	4	4	3	943
4	miR-34	1	1	1	3	2	5	3	3	3	305
5	miR-9	1	4	1	7	7	4	2	3	3	77
6	miR-124	1	1	2	6	5	1	4	3	3	12
7	miR-2	1	8	0	0	0	0	0	0	0	0
8	miR-63	1	0	0	0	0	0	0	0	0	0
9	miR-392	1	0	0	0	0	0	0	0	0	0
**10**	**miR-10**	**0**	**4**	**7**	**15**	**15**	**8**	**5**	**8**	**8**	**313,126**
**11**	**miR-25**	**0**	**2**	**3**	**4**	**4**	**5**	**1**	**4**	**4**	**49,817**
**12**	**miR-7**	**0**	**1**	**2**	**4**	**6**	**3**	**4**	**3**	**3**	**5689**
**13**	**miR-375**	**0**	**1**	**1**	**2**	**2**	**1**	**1**	**1**	**1**	**1846**
**14**	**miR-29**	**0**	**1**	**2**	**3**	**3**	**4**	**4**	**4**	**4**	**1215**
15	miR-184	0	1	1	2	1	1	1	1	1	743
16	miR-133	0	1	2	4	3	4	4	3	3	98
17	miR-190	0	1	1	2	2	1	1	2	2	13
18	miR-210	0	1	1	1	1	1	0	1	1	455
19	miR-193	0	1	1	3	0	1	2	2	2	21
20	miR-33	0	1	0	0	0	2	1	1	2	0
21	miR-314	0	1	0	0	0	0	0	0	0	0
22	miR-31	0	2	0	1	1	1	1	1	1	0
23	miR-263	0	2	0	0	0	0	0	0	0	0
24	miR-219	0	1	0	3	3	1	2	3	3	0
25	miR-216	0	1	0	2	2	1	3	2	2	0

The abbreviation of the animal names used for miRNA nomenclature: Worm (*Caenorhabditis elegans*, cel); Fly (*Drosophila melanogaster*, dme); Common carp (*Cyprinus carpio*, ccr); Zebrafish (*Danio rerio*, dre); Channel catfish (*Ictalurus punctatus*, ipu); Frog (*Xenopus tropicalis*, xtr); Mouse (*Mus musculus*, mmu); Chicken (*Gallus gallus*, gga); Human (*Homo sapiens*, hsa); ccr read represents mRNA read count in the liver of the common carp control sample. The miRNAs in bold represent high abundant expression ones.

**Table 4:** Differentially expressed miRNA species between control and infected samples. The plus (+) value represents up-regulated fold, while the minus (−) value represents down-regulated fold. Two folds difference was set as significant differentially expressed. miRNAs with the absolute value of TPM >3.5 in bold. TPM = miRNA read count/total clean read count × 1,000,000.

No.	Name of miRNA	Control TPM	Infected TPM	Folds
**1**	**miR-365**	**30.03**	**77.28**	**+2.57**
**2**	**miR-196b**	**3.64**	**10.03**	**+2.75**
3	miR-153b	2.12	4.80	+2.26
4	miR-301a	0.76	2.72	+3.58
5	miR-133a-5p	0.30	0.84	+2.75
6	miR-132b	0.61	1.67	+2.75
**7**	**miR-184**	**112.68**	**45.53**	**−2.47**
8	miR-124a	1.21	0.21	−5.76
9	miR-124b	0.61	0.21	−2.91
10	ID_LG1_121431	0.15	2.09	+13.77
11	ID_LG10_120287	2.73	13.16	+4.82
12	ID_000000701_311347	6988.20	26,568.39	+3.80
13	ID_000000130_358988	6988.20	26,568.39	+3.80
14	ID_LG9_186104	1.36	4.59	+3.37
15	ID_LG32_105872	33.37	73.52	+2.20
16	ID_LG42_11512	437.08	924.83	+2.12
17	ID_000001225_235345	3.94	8.35	+2.12
18	ID_LG17_113850	5.76	2.51	−2.30
19	ID_LG40_164682	12.13	5.22	−2.32
20	ID_000000552_212634	11.98	5.01	−2.39
21	ID_LG20_173934	13.95	5.01	−2.78
22	ID_LG11_136853	5.46	1.88	−2.90
23	ID_000000219_202880	3.79	1.25	−3.03
24	ID_000028861_247413	5.16	1.67	−3.09
25	ID_LG30_147265	40.19	9.61	−4.18
26	ID_000001954_374705	40.19	9.61	−4.18
27	ID_000000316_205927	40.19	9.61	−4.18
28	ID_LG27_175722	3.49	0.63	−5.57
29	ID_000028889_299350	1.52	0.00	–
30	ID_000008345_310753	1.52	0.00	–

**Table 5:** Comparison of confirmed miRNA species and families from control and infected samples. Two folds difference was set as significant differentially expressed and is in bold and marked with *.

No.	miRNA Species	miRNA Family	Control TPM	Infected TPM	Infected/Control TPM (miRNA Species)	Infected/Control TPM (miRNA Family)
**1**	**miR-365**	**miR-365**	**30.03**	**77.28**	2.57 *	2.57 *
2	miR-301a	miR-130	0.76	2.72	3.58 *	0.90
3	miR-130b		27.75	26.94	0.97	
4	miR-130c		44.59	37.18	0.83	
5	miR-130a		7.13	5.64	0.79	
6	miR-153b	miR-153	2.12	4.80	2.26 *	1.94
7	miR-153c		8.19	15.25	1.86	
8	miR-196b	miR-196	3.64	10.03	2.75 *	1.77
9	miR-196a		4.25	3.97	0.93	
10	miR-133a-5p	miR-133	0.30	0.84	2.75 *	1.80
11	miR-133a-3p		14.56	25.90	1.78	
12	miR-132b	miR-132	0.61	1.67	2.75 *	1.78
13	miR-132a		3.49	5.64	1.62	
14	miR-9-5p	miR-9	0.46	0.21	0.46	1.13
15	miR-9-3p		11.22	12.95	1.15	
**16**	**miR-184**	**miR-184**	**112.68**	**45.53**	0.40 *	0.40 *
**17**	**miR-124b**	**miR-124**	**0.61**	**0.21**	0.34 *	0.23 *
**18**	**miR-124a**		**1.21**	**0.21**	0.17 *	

## Supplementary Materials

Supplementary materials can be found at http://www.mdpi.com/1422-0067/17/4/566/s1.
