# Supplementary material for: Profilings of MicroRNAs in the Liver of Common Carp (Cyprinus carpio) Infected with Flavobacterium columnare
Source: Int J Mol Sci. 2016 Apr 15;17(4):566. doi: 10.3390/ijms17040566 (PMC4849022; doi:10.3390/ijms17040566)
Supplement: Supplementary file 1 [file ijms-17-00566-s001.pdf]

# Supplementary Materials: Profilings of MicroRNAs in the Liver of Common Carp (*Cyprinus carpio*) Infected with *Flavobacterium columnare*

**Lijuan Zhao, Hong Lu, Qinglei Meng, Jinfu Wang, Weimin Wang, Ling Yang and Li Lin**

**Table S1.** Comparison among the known miRNAs in the liver, spleen, skeletal muscle and pooled 10 tissues of common carp. The ten tissues were: brain, skin, liver, muscle, spleen, head kidney, body kidney, intestine, gill and heart. 8 novel miRNA identified in the liver was marked with +. miRNA with * represents lower expression level. Tissue specific miRNA was shown in bold. The number of specific miRNA detected in the liver, spleen and skeletal muscle was 8, 34, and 19, respectively.

| No. | Liver | Spleen | Skeletal Muscle | Pooled Tissues |
|---|---|---|---|---|
| 1 | ccr-let-7a | ccr-let-7a | cca-let-7a | - |
| 2 | - | - | - | let-7a * |
| 3 | ccr-let-7b | ccr-let-7b | cca-let-7b | let-7b |
| 4 | - | - | - | let-7b * |
| 4 | - | ccr-let-7c | cca-let-7c | - |
| 6 | - | ccr-let-7d | cca-let-7d | - |
| 7 | - | - | **cca-let-7e** | - |
| 8 | - | ccr-let-7f | cca-let-7f | - |
| 9 | ccr-let-7g | ccr-let-7g | cca-let-7g | - |
| 10 | - | - | **cca-let-7h** | - |
| 11 | ccr-let-7i | ccr-let-7i | cca-let-7i | - |
| 12 | ccr-let-7j | ccr-let-7j | cca-let-7j | - |
| 13 | - | **ccr-let-7k** | - | let-7k |
| 14 | ccr-miR-1 | ccr-miR-1 | cca-miR-1 | - |
| 15 | ccr-miR-100 | ccr-miR-100-3p | - | - |
| 16 | - | ccr-miR-100-5p | cca-miR-100 | - |
| 17 | ccr-miR-101a | ccr-miR-101a | cca-miR-101a | - |
| 18 | ccr-miR-101b | ccr-miR-101b | cca-miR-101b | - |
| 19 | ccr-miR-103 | - | cca-miR-103 | - |
| 20 | ccr-miR-107 | ccr-miR-107 | cca-miR-107 | - |
| 21 | - | ccr-miR-10a | cca-miR-10a-5p | - |
| 22 | - | - | **cca-miR-10a-3p** | - |
| 23 | ccr-miR-10b | ccr-miR-10b | cca-miR-10b | - |
| 24 | ccr-miR-10c | ccr-miR-10c | cca-miR-10c | - |
| 25 | ccr-miR-10d | ccr-miR-10d | cca-miR-10d | - |
| 26 | ccr-miR-122 | ccr-miR-122 | cca-miR-122 | - |
| 27 | **ccr-miR-124a** + | - | - | - |
| 28 | **ccr-miR-124b** + | - | - | - |
| 29 | - | **ccr-miR-124c** | - | miR-124 |
| 30 | - | - | - | miR-124 * |
| 31 | - | ccr-miR-125a | cca-miR-125a | - |
| 32 | ccr-miR-125b | ccr-miR-125b | cca-miR-125b | - |
| 33 | ccr-miR-125c | ccr-miR-125c | cca-miR-125c | - |
| 34 | ccr-miR-126-3p | ccr-miR-126-3p | cca-miR-126-3p | - |
| 35 | ccr-miR-126-5p * | ccr-miR-126-5p | cca-miR-126-5p | - |
| 36 | ccr-miR-128 | ccr-miR-128 | cca-miR-128 | - |
| 37 | ccr-miR-129 | ccr-miR-129-3p | - | - |

**Table S1.** *Cont.*

| No. | Liver | Spleen | Skeletal Muscle | Pooled Tissues |
|---|---|---|---|---|
| 38 | - | ccr-miR-129-5p | cca-miR-129 | - |
| 39 | ccr-miR-130a | ccr-miR-130a | cca-miR-130a | - |
| 40 | ccr-miR-130b | ccr-miR-130b-3p | cca-miR-130b | - |
| 41 | - | **ccr-miR-130b-5p** | - | - |
| 42 | ccr-miR-130c | ccr-miR-130c | cca-miR-130c | - |
| 43 | ccr-miR-132a | ccr-miR-132a-3p | cca-miR-132 | miR-132 |
| 44 | - | **ccr-miR-132a-5p** | - | miR-132 * |
| 45 | **ccr-miR-132b** + | - | - | - |
| 46 | ccr-miR-133a-3p | ccr-miR-133a-3p | cca-miR-133a-3p | - |
| 47 | ccr-miR-133a-5p * | ccr-miR-133a-5p | cca-miR-133a-5p | - |
| 48 | - | - | **cca-miR-133b-5p** | - |
| 49 | - | - | **cca-miR-133b-3p** | - |
| 50 | - | - | **cca-miR-133c** | - |
| 51 | - | - | **cca-miR-135a** | - |
| 52 | - | ccr-miR-135b | cca-miR-135b | - |
| 53 | ccr-miR-135c | ccr-miR-135c | cca-miR-135c | - |
| 54 | ccr-miR-137 | ccr-miR-137-3p | cca-miR-137 | miR-137 * |
| 55 | - | **ccr-miR-137-5p** | - | miR-137 |
| 56 | ccr-miR-138 | ccr-miR-138 | cca-miR-138 | - |
| 57 | ccr-miR-139 | ccr-miR-139 | cca-miR-139 | - |
| 58 | - | **ccr-miR-1388-3p** | - | - |
| 59 | - | **ccr-miR-1388-5p** | - | - |
| 60 | ccr-miR-140-3p | ccr-miR-140-3p | cca-miR-140-3p | miR-140 * |
| 61 | ccr-miR-140-5p * | ccr-miR-140-5p | cca-miR-140-5p | miR-140 |
| 62 | - | - | **cca-miR-141** | - |
| 63 | ccr-miR-142-3p + | ccr-miR-142-3p | cca-miR-142a-3p | - |
| 64 | ccr-miR-142-5p + | ccr-miR-142-5p | cca-miR-142a-5p | - |
| 65 | - | - | **cca-miR-142b-5p** | - |
| 66 | ccr-miR-143 | ccr-miR-143 | cca-miR-143 | - |
| 67 | ccr-miR-144 | ccr-miR-144-3p | cca-miR-144 | - |
| 68 | - | **ccr-miR-144-5p** | - | - |
| 69 | - | ccr-miR-145-5p | cca-miR-145 | - |
| 70 | - | **ccr-miR-145-3p** | - | - |
| 71 | ccr-miR-146a | ccr-miR-146a | cca-miR-146a | miR-146a |
| 72 | - | ccr-miR-146b | cca-miR-146b | - |
| 73 | ccr-miR-148 | ccr-miR-148 | cca-miR-148 | - |
| 74 | - | ccr-miR-150 | cca-miR-150 | miR-150 |
| 75 | - | - | - | miR-151-3p |
| 76 | - | ccr-miR-152 | cca-miR-152 | - |
| 77 | ccr-miR-153b | ccr-miR-153b | cca-miR-153b | - |
| 78 | ccr-miR-153c | ccr-miR-153c | cca-miR-153c | - |
| 79 | ccr-miR-155 | ccr-miR-155 | cca-miR-155 | - |
| 80 | ccr-miR-15a | ccr-miR-15a | cca-miR-15a-5p | - |
| 81 | - | - | **cca-miR-15a-3p** | - |
| 82 | ccr-miR-15b | ccr-miR-15b | cca-miR-15b | - |
| 83 | ccr-miR-16a | - | cca-miR-16a | - |
| 84 | ccr-miR-16b | ccr-miR-16b | cca-miR-16b | - |
| 85 | ccr-miR-16c | ccr-miR-16c | cca-miR-16c | - |

**Table S1.** *Cont.*

| No. | Liver | Spleen | Skeletal Muscle | Pooled Tissues |
|---|---|---|---|---|
| 86 | ccr-miR-17-3p * | ccr-miR-17-3p | cca-miR-17-3p | - |
| 87 | ccr-miR-17-5p | ccr-miR-17-5p | cca-miR-17-5p | - |
| 88 | ccr-miR-181a | ccr-miR-181a-3p | cca-miR-181a-3p | - |
| 89 | - | ccr-miR-181a-5p | cca-miR-181a-5p | - |
| 90 | ccr-miR-181b | ccr-miR-181b | cca-miR-181b | - |
| 91 | ccr-miR-181c | ccr-miR-181c | cca-miR-181c | - |
| 92 | - | **ccr-miR-181d** | - | - |
| 93 | - | - | **cca-miR-182-3p** | - |
| 94 | ccr-miR-182-5p | - | cca-miR-182-5p | - |
| 95 | ccr-miR-183 | - | cca-miR-183 | - |
| 96 | ccr-miR-184 | ccr-miR-184 | cca-miR-184 | - |
| 97 | ccr-miR-187 | ccr-miR-187 | cca-miR-187 | - |
| 98 | ccr-miR-18a | ccr-miR-18a | cca-miR-18a | - |
| 99 | cca-miR-18b | - | cca-miR-18b | - |
| 100 | ccr-miR-18c | ccr-miR-18c | cca-miR-18c | - |
| 101 | ccr-miR-190 | ccr-miR-190-3p | - | - |
| 102 | - | ccr-miR-190-5p | cca-miR-190 | - |
| 103 | ccr-miR-192 | ccr-miR-192 | cca-miR-192 | - |
| 104 | ccr-miR-193a | ccr-miR-193a | cca-miR-193a | - |
| 105 | - | ccr-miR-193b | cca-miR-193b | - |
| 106 | ccr-miR-194 | ccr-miR-194 | cca-miR-194 | - |
| 107 | ccr-miR-196a | ccr-miR-196a | cca-miR-196a | - |
| 108 | ccr-miR-196b | ccr-miR-196b | cca-miR-196b | - |
| 109 | ccr-miR-199-3p | ccr-miR-199-3p | cca-miR-199-3p | - |
| 110 | ccr-miR-199-5p | ccr-miR-199-5p | cca-miR-199-5p | - |
| 111 | - | ccr-miR-19a | cca-miR-19a | - |
| 112 | - | ccr-miR-19b | cca-miR-19b-3p | - |
| 113 | - | - | **cca-miR-19b-5p** | - |
| 114 | - | ccr-miR-19c | cca-miR-19c | - |
| 115 | ccr-miR-19d | ccr-miR-19d | cca-miR-19d | - |
| 116 | ccr-miR-200a | ccr-miR-200a | cca-miR-200a | - |
| 117 | ccr-miR-200b | ccr-miR-200b | cca-miR-200b | - |
| 118 | - | ccr-miR-200c | cca-miR-200c | - |
| 119 | - | ccr-miR-202 | cca-miR-202-5p | - |
| 120 | ccr-miR-203a | ccr-miR-203a | cca-miR-203a | - |
| 121 | ccr-miR-203b-3p | - | cca-miR-203b-3p | - |
| 122 | ccr-miR-203b-5p * | - | cca-miR-203b-5p | - |
| 123 | - | ccr-miR-204 | cca-miR-204 | miR-204 |
| 124 | - | - | - | miR-204 * |
| 125 | ccr-miR-205 | - | cca-miR-205 | - |
| 126 | - | ccr-miR-206 | cca-miR-206 | - |
| 127 | ccr-miR-20a-3p * | ccr-miR-20a-3p | cca-miR-20a-3p | - |
| 128 | ccr-miR-20a-5p | ccr-miR-20a-5p | cca-miR-20a-5p | - |
| 129 | - | ccr-miR-20b | cca-miR-20b | - |
| 130 | ccr-miR-21 | ccr-miR-21 | cca-miR-21 | - |
| 131 | ccr-miR-210 | ccr-miR-210-3p | cca-miR-210-3p | - |
| 132 | - | ccr-miR-210-5p | cca-miR-210-5p | - |
| 133 | - | **ccr-miR-212-5p** | - | miR-212-5p |

**Table S1.** *Cont.*

| No. | Liver | Spleen | Skeletal Muscle | Pooled Tissues |
|---|---|---|---|---|
| 134 | - | - | - | miR-212-3p |
| 135 | - | - | - | miR-212a-5p |
| 136 | ccr-miR-214 | ccr-miR-214 | cca-miR-214 | - |
| 137 | - | ccr-miR-216a | cca-miR-216a | - |
| 138 | - | ccr-miR-216b | cca-miR-216b | - |
| 139 | ccr-miR-217 | ccr-miR-217 | cca-miR-217 | - |
| 140 | - | **ccr-miR-2184** | - | - |
| 142 | - | **ccr-miR-2187-3p** | - | - |
| 142 | - | **ccr-miR-2187-5p** | - | - |
| 143 | - | **ccr-miR-2188-5p** | - | - |
| 144 | - | **ccr-miR-2188-3p** | - | - |
| 145 | ccr-miR-218a | ccr-miR-218a | cca-miR-218a | - |
| 146 | ccr-miR-218b | - | cca-miR-218b | - |
| 147 | ccr-miR-221 | ccr-miR-221-3p | cca-miR-221 | - |
| 148 | - | **ccr-miR-221-5p** | - | - |
| 149 | ccr-miR-222 | ccr-miR-222-3p | cca-miR-222 | - |
| 150 | - | **ccr-miR-222-5p** | - | - |
| 151 | - | - | **cca-miR-223** | miR-223 |
| 152 | ccr-miR-22a | ccr-miR-22a-3p | cca-miR-22a | miR-22a |
| 153 | - | **ccr-miR-22a-5p** | - | - |
| 154 | ccr-miR-22b | ccr-miR-22b | cca-miR-22b | miR-22 |
| 155 | ccr-miR-23a | ccr-miR-23a | cca-miR-23a | - |
| 156 | ccr-miR-23b | ccr-miR-23b-3p | cca-miR-23b | - |
| 157 | - | **ccr-miR-23b-5p** | - | - |
| 158 | - | - | - | miR-23c |
| 159 | ccr-miR-24 | ccr-miR-24 | cca-miR-24 | - |
| 160 | - | **ccr-miR-24b-3p** | - | - |
| 161 | - | **ccr-miR-24b-5p** | - | - |
| 162 | ccr-miR-25 | ccr-miR-25 | cca-miR-25 | - |
| 163 | ccr-miR-26a | ccr-miR-26a | cca-miR-26a | miR-26a |
| 164 | - | - | - | miR-26a-1 * |
| 165 | - | - | **cca-miR-26b** | - |
| 166 | - | - | - | miR-26c |
| 167 | ccr-miR-27a | ccr-miR-27a | cca-miR-27a | - |
| 168 | - | ccr-miR-27b-3p | cca-miR-27b | - |
| 169 | - | **ccr-miR-27b-5p** | - | - |
| 170 | ccr-miR-27c-3p | ccr-miR-27c-3p | cca-miR-27c-3p | - |
| 171 | ccr-miR-27c-5p * | ccr-miR-27c-5p | cca-miR-27c-5p | - |
| 172 | cca-miR-27d | - | cca-miR-27d | - |
| 173 | - | ccr-miR-27e | cca-miR-27e | - |
| 174 | ccr-miR-29a | ccr-miR-29a | cca-miR-29a | - |
| 175 | ccr-miR-29b | ccr-miR-29b | cca-miR-29b | - |
| 176 | - | **ccr-miR-29c** | - | - |
| 177 | ccr-miR-301a | ccr-miR-301a-3p | cca-miR-301a | - |
| 178 | - | **ccr-miR-301a-5p** | - | - |
| 179 | - | ccr-miR-301b-3p | cca-miR-301b | - |
| 180 | - | **ccr-miR-301b-5p** | - | - |
| 181 | - | - | **cca-miR-301c** | - |

**Table S1.** *Cont.*

| No. | Liver | Spleen | Skeletal Muscle | Pooled Tissues |
|---|---|---|---|---|
| 182 | - | ccr-miR-30a | cca-miR-30a | - |
| 183 | ccr-miR-30b | ccr-miR-30b | cca-miR-30b | - |
| 184 | - | ccr-miR-30c | cca-miR-30c | - |
| 185 | ccr-miR-30d | ccr-miR-30d | cca-miR-30d | - |
| 186 | - | ccr-miR-30e-5p | cca-miR-30e-5p | - |
| 187 | - | ccr-miR-30e-3p | cca-miR-30e-3p | - |
| 188 | - | ccr-miR-31 | cca-miR-31 | - |
| 189 | ccr-miR-338 | ccr-miR-338 | cca-miR-338 | miR-338-3p |
| 190 | - | - | - | miR-338 * |
| 191 | ccr-miR-34 | ccr-miR-34 | cca-miR-34 | - |
| 192 | ccr-miR-363 | ccr-miR-363 | cca-miR-363 | - |
| 193 | ccr-miR-365 | ccr-miR-365 | cca-miR-365 | - |
| 194 | ccr-miR-375 | ccr-miR-375 | cca-miR-375 | - |
| 195 | ccr-miR-429 | ccr-miR-429 | cca-miR-429 | - |
| 196 | cca-miR-430 | - | cca-miR-430 | miR-430a |
| 197 | - | - | - | miR-430b-1 |
| 198 | - | - | - | miR-430b-2 |
| 199 | - | - | - | miR-430c-1 |
| 200 | - | - | - | miR-430c-2 |
| 201 | - | - | **cca-miR-451** | - |
| 202 | ccr-miR-454a | ccr-miR-454a | cca-miR-454a | - |
| 203 | ccr-miR-454b | ccr-miR-454b | cca-miR-454b | - |
| 204 | ccr-miR-455-3p | ccr-miR-455-3p | - | - |
| 205 | - | ccr-miR-455-5p | cca-miR-455 | - |
| 206 | - | - | **cca-miR-456** | - |
| 207 | ccr-miR-457a | ccr-miR-457a | cca-miR-457a | - |
| 208 | - | ccr-miR-457b-5p | cca-miR-457b | - |
| 209 | ccr-miR-457b | ccr-miR-457b-3p | - | - |
| 210 | - | ccr-miR-458a | cca-miR-458 | - |
| 211 | - | **ccr-miR-458b** | - | - |
| 212 | cca-miR-459-3p | - | cca-miR-459-3p | - |
| 213 | cca-miR-459-5p | - | cca-miR-459-5p | - |
| 214 | cca-miR-460-3p | - | cca-miR-460-3p | - |
| 215 | cca-miR-460-5p | - | cca-miR-460-5p | - |
| 216 | - | - | - | miR-460b-3p |
| 217 | - | - | - | miR-460b-5p |
| 218 | - | ccr-miR-462 | cca-miR-462 | - |
| 219 | ccr-miR-489 | ccr-miR-489 | cca-miR-489 | - |
| 220 | ccr-miR-499 | ccr-miR-499 | cca-miR-499 | miR-499 |
| 221 | - | - | - | miR-499 * |
| 222 | - | - | - | miR-499a-3p |
| 223 | ccr-miR-551 | ccr-miR-551 | - | - |
| 224 | ccr-miR-722 | ccr-miR-722 | cca-miR-722 | - |
| 225 | **ccr-miR-7132** + | - | - | - |
| 226 | **ccr-miR-7133** + | - | - | - |
| 227 | **ccr-miR-722** + | - | - | - |
| 228 | - | **ccr-miR-723** | - | miR-723 * |
| 229 | ccr-miR-724 | ccr-miR-724 | cca-miR-724 | - |

**Table S1.** *Cont.*

| No. | Liver | Spleen | Skeletal Muscle | Pooled Tissues |
|---|---|---|---|---|
| 230 | cca-miR-725 | - | cca-miR-725 | - |
| 231 | ccr-miR-726 | ccr-miR-726 | cca-miR-726 | - |
| 232 | ccr-miR-727-3p | ccr-miR-727-3p | cca-miR-727-3p | miR-727 |
| 233 | ccr-miR-727-5p | ccr-miR-727-5p | cca-miR-727-5p | miR-727 * |
| 234 | ccr-miR-729 | ccr-miR-729 | cca-miR-729 | - |
| 235 | ccr-miR-730 | ccr-miR-730 | cca-miR-730 | - |
| 236 | - | - | **cca-miR-731** | - |
| 237 | ccr-miR-734 | ccr-miR-734 | cca-miR-734 | - |
| 238 | - | **ccr-miR-737** | - | - |
| 239 | - | - | **cca-miR-738** | - |
| 240 | ccr-miR-7a | ccr-miR-7a | cca-miR-7a | miR-7 |
| 241 | - | - | - | miR-7 * |
| 242 | - | - | - | miR-7a-2 * |
| 243 | ccr-miR-7b | ccr-miR-7b | cca-miR-7b | miR-7b |
| 244 | - | ccr-miR-9-5p | cca-miR-9-5p | miR-9a-5p |
| 245 | - | ccr-miR-9-3p | cca-miR-9-3p | miR-9 * |
| 246 | - | - | - | miR-9b-5p |
| 247 | ccr-miR-92a | ccr-miR-92a | cca-miR-92a | - |
| 248 | ccr-miR-92b | ccr-miR-92b | cca-miR-92b | - |
| 249 | ccr-miR-93 | ccr-miR-93 | cca-miR-93 | - |
| 250 | **ccr-miR-9-3p** + | - | - | - |
| 251 | **ccr-miR-9-5p** + | - | - | - |
| 252 | ccr-miR-96 | - | cca-miR-96 | - |
| 253 | ccr-miR-99 | ccr-miR-99 | cca-miR-99 | - |
| 254 | - | **ccr-miR-3120** | - | - |
| 255 | - | **ccr-miR-4837** | - | - |
| 256 | - | **ccr-miR-5109** | - | - |
| 257 | - | **ccr-miR-6651** | - | - |
| 258 | - | **ccr-miR-696** | - | - |
| 259 | - | - | - | miR-1601 |
| 260 | - | - | - | miR-1623 |
| 261 | - | - | - | miR-1754 |
| 262 | - | - | - | miR-2130 |
| 263 | - | - | - | miR-297 |
| 264 | - | - | - | miR-3065-3p |
| 265 | - | - | - | miR-3065-5p |
| 266 | - | - | - | miR-306a * |
| 267 | - | - | - | miR-306b |
| 268 | - | - | - | miR-3112 * |
| 269 | - | - | - | miR-3167 |
| 270 | - | - | - | miR-3217 |
| 271 | - | - | - | miR-3384 |
| 272 | - | - | - | miR-3529 |
| 273 | - | - | - | miR-3596 |
| 274 | - | - | - | miR-3597-3p |
| 275 | - | - | - | miR-3597-5p |
| 276 | - | - | - | miR-3600 |
| 277 | - | - | - | miR-3611 |

**Table S1.** *Cont.*

| No. | Liver | Spleen | Skeletal Muscle | Pooled Tissues |
|---|---|---|---|---|
| 278 | - | - | - | miR-3686 |
| 279 | - | - | - | miR-4072-3p |
| 280 | - | - | - | miR-4143-5p |
| 281 | - | - | - | miR-4204-3p |
| 282 | - | - | - | miR-4453 |
| 283 | - | - | - | miR-4666-3p |
| 284 | - | - | - | miR-4666-5p |
| 285 | - | - | - | miR-466c * |
| 286 | - | - | - | miR-5113 |
| 287 | - | - | - | miR-541 |
| 288 | - | - | - | miR-669 |
| 289 | - | - | - | miR-669n |
| 290 | Total: 142 | Total: 193 | Total: 188 | Total: 80 |

**Table S2.** Comparison of miRNA families of common carp and other represented animals. Ccr count reads with TPM more than 1000 were shown in bold.

| No. | miRNA Family | cel | dme | ccr | dre | ipu | xtr | gga | mmu | hsa | ccr Read |
|---|---|---|---|---|---|---|---|---|---|---|---|
| **1** | **miR-let_7** | **1** | **1** | **5** | **18** | **21** | **9** | **11** | **12** | **12** | **185,356** |
| **2** | **miR-8** | **1** | **1** | **3** | **6** | **6** | **3** | **3** | **5** | **5** | **41,287** |
| 3 | miR-1 | 1 | 1 | 2 | 4 | 3 | 4 | 4 | 4 | 3 | 943 |
| 4 | miR-34 | 1 | 1 | 1 | 3 | 2 | 5 | 3 | 3 | 3 | 305 |
| 5 | miR-9 | 1 | 4 | 1 | 7 | 7 | 4 | 2 | 3 | 3 | 77 |
| 6 | miR-124 | 1 | 1 | 2 | 6 | 5 | 1 | 4 | 3 | 3 | 12 |
| 7 | miR-2 | 1 | 8 | 0 | 0 | 0 | 0 | 0 | 0 | 0 | 0 |
| 8 | miR-63 | 1 | 0 | 0 | 0 | 0 | 0 | 0 | 0 | 0 | 0 |
| 9 | miR-392 | 1 | 0 | 0 | 0 | 0 | 0 | 0 | 0 | 0 | 0 |
| **10** | **miR-10** | **0** | **4** | **7** | **15** | **15** | **8** | **5** | **8** | **8** | **313,126** |
| **11** | **miR-25** | **0** | **2** | **3** | **4** | **4** | **5** | **1** | **4** | **4** | **49,817** |
| **12** | **miR-7** | **0** | **1** | **2** | **4** | **6** | **3** | **4** | **3** | **3** | **5689** |
| **13** | **miR-375** | **0** | **1** | **1** | **2** | **2** | **1** | **1** | **1** | **1** | **1846** |
| **14** | **miR-29** | **0** | **1** | **2** | **3** | **3** | **4** | **4** | **4** | **4** | **1215** |
| 15 | miR-184 | 0 | 1 | 1 | 2 | 1 | 1 | 1 | 1 | 1 | 743 |
| 16 | miR-133 | 0 | 1 | 2 | 4 | 3 | 4 | 4 | 3 | 3 | 98 |
| 17 | miR-190 | 0 | 1 | 1 | 2 | 2 | 1 | 1 | 2 | 2 | 13 |
| 18 | miR-210 | 0 | 1 | 1 | 1 | 1 | 1 | 0 | 1 | 1 | 455 |
| 19 | miR-193 | 0 | 1 | 1 | 3 | 0 | 1 | 2 | 2 | 2 | 21 |
| 20 | miR-33 | 0 | 1 | 0 | 0 | 0 | 2 | 1 | 1 | 2 | 0 |
| 21 | miR-314 | 0 | 1 | 0 | 0 | 0 | 0 | 0 | 0 | 0 | 0 |
| 22 | miR-31 | 0 | 2 | 0 | 1 | 1 | 1 | 1 | 1 | 1 | 0 |
| 23 | miR-263 | 0 | 2 | 0 | 0 | 0 | 0 | 0 | 0 | 0 | 0 |
| 24 | miR-219 | 0 | 1 | 0 | 3 | 3 | 1 | 2 | 3 | 3 | 0 |
| 25 | miR-216 | 0 | 1 | 0 | 2 | 2 | 1 | 3 | 2 | 2 | 0 |
| **26** | **miR-22** | **0** | **0** | **2** | **2** | **2** | **1** | **1** | **1** | **1** | **1,406,179** |
| **27** | **miR-146** | **0** | **0** | **1** | **2** | **2** | **2** | **3** | **2** | **2** | **373,505** |
| **28** | **miR-192** | **0** | **0** | **1** | **1** | **1** | **2** | **1** | **2** | **2** | **259,866** |
| **29** | **miR-148** | **0** | **0** | **1** | **2** | **2** | **2** | **1** | **3** | **3** | **218,905** |
| **30** | **miR-21** | **0** | **0** | **1** | **2** | **2** | **0** | **1** | **1** | **1** | **197,102** |
| **31** | **miR-126** | **0** | **0** | **2** | **2** | **2** | **1** | **1** | **2** | **1** | **195,299** |
| **32** | **miR-143** | **0** | **0** | **1** | **2** | **1** | **1** | **0** | **1** | **1** | **143,292** |
| **33** | **miR-26** | **0** | **0** | **1** | **4** | **4** | **2** | **1** | **3** | **3** | **142,777** |
| **34** | **miR-101** | **0** | **0** | **2** | **2** | **2** | **2** | **2** | **2** | **2** | **121,627** |
| **35** | **miR-199** | **0** | **0** | **2** | **3** | **4** | **2** | **2** | **3** | **3** | **113,615** |
| **36** | **miR-17** | **0** | **0** | **7** | **8** | **4** | **8** | **6** | **8** | **8** | **53,911** |
| **37** | **miR-30** | **0** | **0** | **2** | **5** | **5** | **6** | **6** | **6** | **6** | **32,153** |
| **38** | **miR-194** | **0** | **0** | **1** | **2** | **1** | **2** | **1** | **2** | **2** | **31,383** |
| **39** | **miR-221** | **0** | **0** | **2** | **3** | **4** | **2** | **3** | **2** | **2** | **21,959** |
| **40** | **miR-181** | **0** | **0** | **3** | **5** | **8** | **4** | **4** | **6** | **6** | **21,556** |
| **41** | **miR-15** | **0** | **0** | **7** | **9** | **5** | **6** | **6** | **5** | **5** | **20,235** |
| **42** | **miR-140** | **0** | **0** | **2** | **1** | **1** | **1** | **1** | **1** | **1** | **12,291** |
| **43** | **miR-128** | **0** | **0** | **1** | **2** | **2** | **2** | **2** | **2** | **2** | **9999** |
| **44** | **miR-103** | **0** | **0** | **2** | **3** | **4** | **3** | **3** | **3** | **5** | **9853** |
| **45** | **miR-23** | **0** | **0** | **2** | **5** | **5** | **3** | **1** | **2** | **2** | **3326** |
| **46** | **miR-27** | **0** | **0** | **3** | **6** | **5** | **4** | **1** | **2** | **2** | **2943** |
| **47** | **miR-144** | **0** | **0** | **1** | **1** | **1** | **1** | **1** | **1** | **1** | **2131** |

**Table S2.** *Cont.*

| No. | miRNA Family | cel | dme | ccr | dre | ipu | xtr | gga | mmu | hsa | ccr Read |
|---|---|---|---|---|---|---|---|---|---|---|---|
| **48** | **miR-24** | **0** | **0** | **1** | **5** | **4** | **2** | **1** | **2** | **2** | **1955** |
| **49** | **miR-142** | **0** | **0** | **2** | **2** | **2** | **2** | **1** | **2** | **1** | **1640** |
| 50 | miR-455 | 0 | 0 | 1 | 2 | 2 | 1 | 1 | 1 | 1 | 617 |
| 51 | miR-130 | 0 | 0 | 4 | 7 | 4 | 5 | 5 | 4 | 4 | 529 |
| 52 | miR-214 | 0 | 0 | 1 | 1 | 1 | 1 | 1 | 1 | 2 | 494 |
| 53 | miR-203 | 0 | 0 | 3 | 2 | 3 | 1 | 1 | 1 | 2 | 483 |
| 54 | miR-155 | 0 | 0 | 1 | 1 | 1 | 1 | 1 | 1 | 1 | 363 |
| 55 | miR-19 | 0 | 0 | 1 | 4 | 3 | 3 | 2 | 3 | 3 | 347 |
| 56 | miR-218 | 0 | 0 | 2 | 3 | 3 | 2 | 2 | 2 | 2 | 321 |
| 57 | miR-365 | 0 | 0 | 1 | 3 | 2 | 1 | 2 | 2 | 2 | 198 |
| 58 | miR-187 | 0 | 0 | 1 | 2 | 1 | 1 | 1 | 1 | 1 | 82 |
| 59 | miR-153 | 0 | 0 | 2 | 3 | 1 | 2 | 1 | 1 | 2 | 68 |
| 60 | miR-196 | 0 | 0 | 2 | 5 | 4 | 2 | 5 | 3 | 3 | 52 |
| 61 | miR-205 | 0 | 0 | 1 | 1 | 1 | 2 | 2 | 1 | 1 | 36 |
| 62 | miR-135 | 0 | 0 | 1 | 4 | 4 | 2 | 3 | 3 | 3 | 36 |
| 63 | miR-489 | 0 | 0 | 1 | 1 | 1 | 1 | 1 | 1 | 1 | 26 |
| 64 | miR-499 | 0 | 0 | 1 | 1 | 2 | 1 | 1 | 1 | 1 | 21 |
| 65 | miR-183 | 0 | 0 | 1 | 1 | 1 | 1 | 1 | 1 | 1 | 16 |
| 66 | miR-138 | 0 | 0 | 1 | 1 | 2 | 1 | 2 | 2 | 2 | 11 |
| 67 | miR-139 | 0 | 0 | 1 | 1 | 1 | 1 | 0 | 1 | 1 | 452 |
| 68 | miR-338 | 0 | 0 | 1 | 2 | 3 | 2 | 0 | 1 | 1 | 399 |
| 69 | miR-129 | 0 | 0 | 1 | 2 | 5 | 2 | 0 | 3 | 2 | 41 |
| 70 | miR-132 | 0 | 0 | 2 | 4 | 4 | 2 | 0 | 2 | 2 | 27 |
| 71 | miR-182 | 0 | 0 | 2 | 1 | 1 | 1 | 0 | 1 | 1 | 15 |
| 72 | miR-96 | 0 | 0 | 1 | 1 | 1 | 1 | 0 | 1 | 1 | 2 |
| 73 | miR-454 | 0 | 0 | 2 | 2 | 1 | 0 | 1 | 0 | 1 | 1891 |
| 74 | miR-460 | 0 | 0 | 2 | 2 | 2 | 0 | 2 | 0 | 0 | 461 |
| 75 | miR-551 | 0 | 0 | 1 | 0 | 0 | 0 | 1 | 1 | 2 | 26 |
| 76 | miR-459 | 0 | 0 | 2 | 1 | 1 | 0 | 0 | 0 | 0 | 12 |
| 77 | miR-430 | 0 | 0 | 1 | 59 | 0 | 0 | 0 | 0 | 0 | 2 |
| 78 | miR-725 | 0 | 0 | 1 | 2 | 0 | 0 | 0 | 0 | 0 | 197 |
| 79 | miR-204 | 0 | 0 | 0 | 2 | 3 | 2 | 3 | 2 | 2 | 0 |
| 80 | miR-458 | 0 | 0 | 0 | 1 | 1 | 0 | 2 | 0 | 0 | 0 |
| 81 | miR-145 | 0 | 0 | 0 | 1 | 1 | 1 | 0 | 1 | 1 | 0 |
| 82 | miR-1388 | 0 | 0 | 0 | 1 | 1 | 0 | 0 | 0 | 0 | 0 |
| 83 | miR-202 | 0 | 0 | 0 | 1 | 1 | 2 | 1 | 1 | 1 | 0 |
| 84 | miR-2188 | 0 | 0 | 0 | 1 | 1 | 1 | 1 | 0 | 0 | 0 |
| 85 | miR-150 | 0 | 0 | 0 | 1 | 1 | 1 | 0 | 1 | 1 | 0 |
| 86 | miR-456 | 0 | 0 | 0 | 1 | 1 | 0 | 1 | 0 | 0 | 0 |
| 87 | miR-3618 | 0 | 0 | 0 | 0 | 1 | 0 | 0 | 1 | 1 | 0 |
| 88 | miR-320 | 0 | 0 | 0 | 0 | 0 | 0 | 0 | 1 | 8 | 0 |
| 89 | miR-154 | 0 | 0 | 0 | 0 | 0 | 0 | 0 | 13 | 19 | 0 |
| 90 | miR-4662 | 0 | 0 | 0 | 0 | 0 | 0 | 0 | 0 | 2 | 0 |
| 91 | miR-548 | 0 | 0 | 0 | 0 | 0 | 0 | 0 | 0 | 74 | 0 |
| **92** | **miR-122** | **0** | **0** | **1** | **0** | **0** | **0** | **0** | **0** | **0** | **1,028,308** |
| **93** | **miR-722** | **0** | **0** | **1** | **0** | **0** | **0** | **0** | **0** | **0** | **60,888** |
| **94** | **miR-217** | **0** | **0** | **1** | **0** | **0** | **0** | **0** | **0** | **0** | **44,611** |
| **95** | **miR-363** | **0** | **0** | **1** | **0** | **0** | **0** | **0** | **0** | **0** | **8475** |

**Table S2.** *Cont.*

| No. | miRNA Family | cel | dme | ccr | dre | ipu | xtr | gga | mmu | hsa | ccr Read |
|---|---|---|---|---|---|---|---|---|---|---|---|
| **96** | **miR-724** | **0** | **0** | **1** | **0** | **0** | **0** | **0** | **0** | **0** | **1054** |
| 97 | miR-137 | 0 | 0 | 1 | 0 | 0 | 0 | 0 | 0 | 0 | 51 |
| 98 | miR-726 | 0 | 0 | 1 | 0 | 0 | 0 | 0 | 0 | 0 | 36 |
| 99 | miR-727 | 0 | 0 | 2 | 0 | 0 | 0 | 0 | 0 | 0 | 35 |
| 100 | miR-729 | 0 | 0 | 1 | 0 | 0 | 0 | 0 | 0 | 0 | 1 |
| 101 | miR-734 | 0 | 0 | 1 | 0 | 0 | 0 | 0 | 0 | 0 | 1 |

The abbreviation of the animal names used for miRNA nomenclature. Worm (*Caenorhabditis elegans*, cel); Fly (*Drosophila melanogaster*, dme); Common carp (*Cyprinus carpio*, ccr); Zebrafish (*Danio rerio*, dre); Channel catfish (*Ictalurus punctatus*, ipu); Frog (*Xenopus tropicalis*, xtr); Mouse (*Mus musculus*, mmu); Chicken (*Gallus gallus*, gga); Human (*Homo sapiens*, hsa); miRNAs in bold represent high abundant expression ones.

**Table S3.** Comparison of the abundance of identified miRNA species from control and infected samples. 2 folds difference was set as significant differentially expressed. The differentially expressed miRNAs were shown in bold and marked with *.

| No. | miRNA Species | Control Read Count | Infected Read Count | Control TPM | Infected TPM | Infected/ Control TPM |
|---|---|---|---|---|---|---|
| **1** | **miR-301a** | **5** | **13** | **0.76** | **2.72** | **3.58 *** |
| **2** | **miR-132b** | **4** | **8** | **0.61** | **1.67** | **2.75 *** |
| **3** | **miR-133a-5p** | **2** | **4** | **0.30** | **0.84** | **2.75 *** |
| **4** | **miR-196b** | **24** | **48** | **3.64** | **10.03** | **2.75 *** |
| **5** | **miR-365** | **198** | **370** | **30.03** | **77.28** | **2.57 *** |
| **6** | **miR-153b** | **14** | **23** | **2.12** | **4.80** | **2.26 *** |
| 7 | miR-155 | 363 | 506 | 55.05 | 105.68 | 1.92 |
| 8 | miR-190 | 13 | 18 | 1.97 | 3.76 | 1.91 |
| 9 | miR-153c | 54 | 73 | 8.19 | 15.25 | 1.86 |
| 10 | miR-1 | 943 | 1238 | 143.02 | 258.57 | 1.81 |
| 11 | miR-133a-3p | 96 | 124 | 14.56 | 25.90 | 1.78 |
| 12 | miR-205 | 36 | 46 | 5.46 | 9.61 | 1.76 |
| 13 | miR-182-5p | 15 | 19 | 2.27 | 3.97 | 1.74 |
| 14 | miR-146a | 373,505 | 458,074 | 56,645.85 | 95,673.10 | 1.69 |
| 15 | miR-21 | 197,102 | 234,054 | 29,892.53 | 48,884.40 | 1.64 |
| 16 | miR-132a | 23 | 27 | 3.49 | 5.64 | 1.62 |
| 17 | miR-19d | 347 | 401 | 52.63 | 83.75 | 1.59 |
| 18 | miR-727-3p | 32 | 35 | 4.85 | 7.31 | 1.51 |
| 19 | miR-7b | 2305 | 2517 | 349.58 | 525.70 | 1.50 |
| 20 | miR-7132 | 23 | 25 | 3.49 | 5.22 | 1.50 |
| 21 | miR-93 | 2745 | 2902 | 416.31 | 606.11 | 1.46 |
| 22 | miR-457a | 614 | 633 | 93.12 | 132.21 | 1.42 |
| 23 | miR-7a | 3384 | 3430 | 513.22 | 716.39 | 1.40 |
| 24 | miR-183 | 16 | 16 | 2.43 | 3.34 | 1.38 |
| 25 | miR-430 | 2 | 2 | 0.30 | 0.42 | 1.38 |
| 26 | miR-734 | 1 | 1 | 0.15 | 0.21 | 1.38 |
| 27 | miR-454a | 213 | 209 | 32.30 | 43.65 | 1.35 |
| 28 | miR-10c | 18,262 | 17,663 | 2769.62 | 3689.09 | 1.33 |
| 29 | miR-138 | 11 | 10 | 1.67 | 2.09 | 1.25 |
| 30 | miR-375 | 1846 | 1639 | 279.96 | 342.32 | 1.22 |
| 31 | let-7i | 26,758 | 23,339 | 4058.12 | 4874.57 | 1.20 |
| 32 | miR-16b | 13,533 | 11,784 | 2052.42 | 2461.20 | 1.20 |
| 33 | miR-29a | 696 | 604 | 105.56 | 126.15 | 1.20 |
| 34 | miR-27c-5p | 85 | 74 | 12.89 | 15.46 | 1.20 |
| 35 | miR-10b | 30,202 | 25,771 | 4580.44 | 5382.52 | 1.18 |
| 36 | miR-23b | 822 | 705 | 124.66 | 147.25 | 1.18 |
| 37 | miR-23a | 2504 | 2123 | 379.76 | 443.41 | 1.17 |
| 38 | miR-222 | 6118 | 5142 | 927.86 | 1073.96 | 1.16 |
| 39 | miR-724 | 1054 | 890 | 159.85 | 185.88 | 1.16 |

**Table S3.** *Cont.*

| No. | miRNA Species | Control Read Count | Infected Read Count | Control TPM | Infected TPM | Infected/ Control TPM |
|---|---|---|---|---|---|---|
| 40 | miR-16c | 2469 | 2066 | 374.45 | 431.50 | 1.15 |
| 41 | miR-9-3p | 74 | 62 | 11.22 | 12.95 | 1.15 |
| 42 | miR-135c | 36 | 30 | 5.46 | 6.27 | 1.15 |
| 43 | miR-16a | 2306 | 1915 | 349.73 | 399.97 | 1.14 |
| 44 | miR-22b | 160 | 132 | 24.27 | 27.57 | 1.14 |
| 45 | miR-20a-5p | 37,757 | 30,938 | 5726.23 | 6461.69 | 1.13 |
| 46 | miR-181b | 3035 | 2488 | 460.29 | 519.64 | 1.13 |
| 47 | miR-140-5p | 1975 | 1619 | 299.53 | 338.14 | 1.13 |
| 48 | miR-29b | 519 | 427 | 78.71 | 89.18 | 1.13 |
| 49 | miR-203b-3p | 134 | 109 | 20.32 | 22.77 | 1.12 |
| 50 | miR-454b | 1678 | 1358 | 254.49 | 283.63 | 1.11 |
| 51 | miR-15a | 624 | 502 | 94.64 | 104.85 | 1.11 |
| 52 | miR-30b | 2224 | 1778 | 337.29 | 371.35 | 1.10 |
| 53 | miR-725 | 197 | 158 | 29.88 | 33.00 | 1.10 |
| 54 | miR-217 | 44,611 | 35,408 | 6765.71 | 7395.30 | 1.09 |
| 55 | miR-218a | 298 | 236 | 45.19 | 49.29 | 1.09 |
| 56 | let-7g | 16,993 | 13,174 | 2577.16 | 2751.51 | 1.07 |
| 57 | miR-92a | 49,709 | 38,329 | 7538.88 | 8005.38 | 1.06 |
| 58 | miR-221 | 15,841 | 12,131 | 2402.45 | 2533.67 | 1.05 |
| 59 | miR-457b | 634 | 484 | 96.15 | 101.09 | 1.05 |
| 60 | miR-199-5p | 93,165 | 70,574 | 14,129.42 | 14,740.05 | 1.04 |
| 61 | miR-10d | 431 | 324 | 65.37 | 67.67 | 1.04 |
| 62 | miR-17-5p | 10,617 | 7916 | 1610.18 | 1653.33 | 1.03 |
| 63 | miR-27c-3p | 1651 | 1230 | 250.39 | 256.90 | 1.03 |
| 64 | miR-181c | 122 | 91 | 18.50 | 19.01 | 1.03 |
| 65 | miR-455 | 617 | 456 | 93.57 | 95.24 | 1.02 |
| 66 | miR-722 | 60,888 | 44,697 | 9234.29 | 9335.39 | 1.01 |
| 67 | miR-148 | 218,905 | 159,083 | 33,199.18 | 33,225.99 | 1.00 |
| 68 | miR-30d | 29,929 | 21,824 | 4539.04 | 4558.15 | 1.00 |
| 69 | miR-107 | 5572 | 4052 | 845.05 | 846.30 | 1.00 |
| 70 | miR-17-3p | 58 | 42 | 8.80 | 8.77 | 1.00 |
| 71 | miR-103 | 4281 | 3072 | 649.26 | 641.62 | 0.99 |
| 72 | miR-125c | 1543 | 1114 | 234.01 | 232.67 | 0.99 |
| 73 | miR-203a | 348 | 251 | 52.78 | 52.42 | 0.99 |
| 74 | miR-142-3p | 926 | 662 | 140.44 | 138.27 | 0.98 |
| 75 | miR-499 | 21 | 15 | 3.18 | 3.13 | 0.98 |
| 76 | miR-26a | 142,777 | 100,747 | 21,653.59 | 21,041.97 | 0.97 |
| 77 | miR-99 | 45,479 | 31,931 | 6897.36 | 6669.09 | 0.97 |
| 78 | miR-130b | 183 | 129 | 27.75 | 26.94 | 0.97 |
| 79 | miR-92b | 108 | 76 | 16.38 | 15.87 | 0.97 |
| 80 | let-7a | 117,306 | 81,643 | 17,790.65 | 17,051.91 | 0.96 |
| 81 | miR-187 | 82 | 57 | 12.44 | 11.91 | 0.96 |
| 82 | miR-140-3p | 10,316 | 7150 | 1564.53 | 1493.35 | 0.95 |
| 83 | miR-730 | 397 | 275 | 60.21 | 57.44 | 0.95 |
| 84 | let-7j | 12,203 | 8361 | 1850.71 | 1746.27 | 0.94 |
| 85 | miR-126-5p | 1950 | 1326 | 295.74 | 276.95 | 0.94 |
| 86 | miR-129 | 41 | 28 | 6.22 | 5.85 | 0.94 |
| 87 | miR-126-3p | 193,349 | 130,881 | 29,323.35 | 27,335.74 | 0.93 |
| 88 | miR-101b | 5993 | 4045 | 908.90 | 844.84 | 0.93 |
| 89 | miR-196a | 28 | 19 | 4.25 | 3.97 | 0.93 |
| 90 | miR-192 | 259,866 | 173,991 | 39,411.33 | 36,339.67 | 0.92 |
| 91 | miR-210 | 455 | 305 | 69.01 | 63.70 | 0.92 |
| 92 | miR-459-5p | 3 | 2 | 0.46 | 0.42 | 0.92 |
| 93 | miR-727-5p | 3 | 2 | 0.46 | 0.42 | 0.92 |
| 94 | let-7b | 12,096 | 7994 | 1834.48 | 1669.62 | 0.91 |
| 95 | miR-139 | 452 | 299 | 68.55 | 62.45 | 0.91 |
| 96 | miR-200b | 11,950 | 7852 | 1812.34 | 1639.96 | 0.90 |
| 97 | miR-18b | 14 | 9 | 2.12 | 1.88 | 0.89 |
| 98 | miR-199-3p | 20,450 | 13,135 | 3101.45 | 2743.37 | 0.88 |
| 99 | miR-25 | 5635 | 3568 | 854.61 | 745.21 | 0.87 |

**Table S3.** *Cont.*

| No. | miRNA Species | Control Read Count | Infected Read Count | Control TPM | Infected TPM | Infected/ Control TPM |
|---|---|---|---|---|---|---|
| 100 | miR-22a | 1,406,019 | 877,060 | 213,237.15 | 183,182.30 | 0.86 |
| 101 | miR-181a | 18,399 | 11,503 | 2790.40 | 2402.51 | 0.86 |
| 102 | miR-137 | 51 | 32 | 7.73 | 6.68 | 0.86 |
| 103 | miR-125b | 12,371 | 7665 | 1876.19 | 1600.91 | 0.85 |
| 104 | miR-18a | 60 | 37 | 9.10 | 7.73 | 0.85 |
| 105 | miR-363 | 8475 | 5087 | 1285.32 | 1062.47 | 0.83 |
| 106 | miR-130c | 294 | 178 | 44.59 | 37.18 | 0.83 |
| 107 | miR-194 | 31,383 | 18,480 | 4759.55 | 3859.72 | 0.81 |
| 108 | miR-100 | 204,838 | 117,144 | 31,065.78 | 24,466.64 | 0.79 |
| 109 | miR-130a | 47 | 27 | 7.13 | 5.64 | 0.79 |
| 110 | miR-193a | 21 | 12 | 3.18 | 2.51 | 0.79 |
| 111 | miR-7133 | 179 | 102 | 27.15 | 21.30 | 0.78 |
| 112 | miR-218b | 23 | 13 | 3.49 | 2.72 | 0.78 |
| 113 | miR-214 | 494 | 275 | 74.92 | 57.44 | 0.77 |
| 114 | miR-20a-3p | 135 | 75 | 20.47 | 15.66 | 0.77 |
| 115 | miR-459-3p | 9 | 5 | 1.36 | 1.04 | 0.77 |
| 116 | miR-200a | 28,563 | 15,772 | 4331.87 | 3294.13 | 0.76 |
| 117 | miR-144 | 2131 | 1174 | 323.19 | 245.20 | 0.76 |
| 118 | miR-24 | 1955 | 1079 | 296.50 | 225.36 | 0.76 |
| 119 | miR-429 | 774 | 429 | 117.39 | 89.60 | 0.76 |
| 120 | miR-122 | 1,028,308 | 558,847 | 155,953.41 | 116,720.50 | 0.75 |
| 121 | miR-34 | 305 | 164 | 46.26 | 34.25 | 0.74 |
| 122 | miR-128 | 9999 | 5291 | 1516.45 | 1105.08 | 0.73 |
| 123 | miR-101a | 115,634 | 60,318 | 17,537.08 | 12,597.99 | 0.72 |
| 124 | miR-460-5p | 63 | 33 | 9.55 | 6.89 | 0.72 |
| 125 | miR-143 | 143,292 | 73,572 | 21,731.70 | 15,366.21 | 0.71 |
| 126 | miR-338 | 399 | 206 | 60.51 | 43.03 | 0.71 |
| 127 | miR-551 | 26 | 13 | 3.94 | 2.72 | 0.69 |
| 128 | miR-27a | 517 | 251 | 78.41 | 52.42 | 0.67 |
| 129 | miR-18c | 2525 | 1198 | 382.94 | 250.21 | 0.65 |
| 130 | miR-142-5p | 714 | 335 | 108.29 | 69.97 | 0.65 |
| 131 | miR-15b | 55 | 26 | 8.34 | 5.43 | 0.65 |
| 132 | miR-726 | 36 | 16 | 5.46 | 3.34 | 0.61 |
| 133 | miR-489 | 26 | 11 | 3.94 | 2.30 | 0.58 |
| 134 | miR-27d | 690 | 258 | 104.65 | 53.89 | 0.51 |
| **135** | **miR-9-5p** | **3** | **1** | **0.46** | **0.21** | **0.46 *** |
| **136** | **miR-184** | **743** | **218** | **112.68** | **45.53** | **0.40 *** |
| **137** | **miR-124b** | **4** | **1** | **0.61** | **0.21** | **0.34 *** |
| **138** | **miR-124a** | **8** | **1** | **1.21** | **0.21** | **0.17 *** |
| 139 | miR-96 | 2 | 0 | 0.30 | 0.00 | 0.00 |
| 140 | miR-203b-5p | 1 | 0 | 0.15 | 0.00 | 0.00 |
| 141 | miR-460-3p | 1 | 0 | 0.15 | 0.00 | 0.00 |
| 142 | miR-729 | 1 | 0 | 0.15 | 0.00 | 0.00 |

**Table S4.** Comparison of the miRNA family from infected and non-infected samples. The differentially expressed miRNA families were shown in bold and marked with *. (2 folds difference was set as significant differentially expressed).

| No. | Family | Control (TPM) | Infected (TPM) | Infected/Control |
|---|---|---|---|---|
| **1** | **miR-365** | **30.03** | **77.28** | **2.57 *** |
| 2 | miR-153 | 10.31 | 20.05 | 1.94 |
| 3 | miR-155 | 55.05 | 105.68 | 1.92 |
| 4 | miR-190 | 1.97 | 3.76 | 1.91 |
| 5 | miR-1 | 143.02 | 258.57 | 1.81 |
| 6 | miR-133 | 14.86 | 26.73 | 1.80 |
| 7 | miR-132 | 4.10 | 7.31 | 1.79 |
| 8 | miR-196 | 7.89 | 13.99 | 1.77 |
| 9 | miR-205 | 5.46 | 9.61 | 1.76 |
| 10 | miR-182 | 2.28 | 3.97 | 1.74 |
| 11 | miR-146 | 56,645.85 | 95,673.10 | 1.69 |
| 12 | miR-21 | 29,892.53 | 48,884.40 | 1.64 |
| 13 | miR-19 | 52.63 | 83.75 | 1.59 |
| 14 | miR-7132 | 3.49 | 5.22 | 1.50 |
| 15 | miR-727 | 5.31 | 7.73 | 1.46 |
| 16 | miR-7 | 862.80 | 1242.09 | 1.44 |
| 17 | miR-183 | 2.43 | 3.34 | 1.38 |
| 18 | miR-430 | 0.30 | 0.42 | 1.38 |
| 19 | miR-734 | 0.15 | 0.21 | 1.38 |
| 20 | miR-138 | 1.67 | 2.09 | 1.25 |
| 21 | miR-375 | 279.97 | 342.32 | 1.22 |
| 22 | miR-15 | 3068.85 | 3636.24 | 1.18 |
| 23 | miR-23 | 504.42 | 590.66 | 1.17 |
| 24 | miR-29 | 184.27 | 215.33 | 1.17 |
| 25 | miR-724 | 159.85 | 185.89 | 1.16 |
| 26 | miR-135 | 5.46 | 6.27 | 1.15 |
| 27 | miR-454 | 286.79 | 327.28 | 1.14 |
| 28 | miR-9 | 11.68 | 13.16 | 1.13 |
| 29 | miR-17 | 8176.15 | 9005.40 | 1.10 |
| 30 | miR-725 | 29.88 | 33.00 | 1.10 |
| 31 | miR-217 | 6765.71 | 7395.30 | 1.09 |
| 32 | miR-221 | 3330.31 | 3607.63 | 1.08 |
| 33 | miR-218 | 48.68 | 52.01 | 1.07 |
| 34 | miR-25 | 8409.86 | 8766.46 | 1.04 |
| 35 | miR-203 | 73.25 | 75.19 | 1.03 |
| 36 | miR-455 | 93.57 | 95.24 | 1.02 |
| 37 | miR-199 | 17,230.88 | 17,483.42 | 1.01 |
| 38 | miR-722 | 9234.29 | 9335.39 | 1.01 |
| 39 | miR-30 | 4876.33 | 4929.50 | 1.01 |
| 40 | miR-148 | 33,199.18 | 33,225.99 | 1.00 |
| 41 | let-7 | 28,111.13 | 28,093.90 | 1.00 |
| 42 | miR-103 | 1494.31 | 1487.92 | 1.00 |
| 43 | miR-140 | 1864.06 | 1831.49 | 0.98 |
| 44 | miR-499 | 3.19 | 3.13 | 0.98 |
| 45 | miR-26 | 21,653.59 | 21,041.97 | 0.97 |
| 46 | miR-187 | 12.44 | 11.91 | 0.96 |

**Table S4.** *Cont.*

| No. | Family | Control (TPM) | Infected (TPM) | Infected/Control |
|---|---|---|---|---|
| 47 | miR-129 | 6.22 | 5.85 | 0.94 |
| 48 | miR-126 | 29,619.09 | 27,612.69 | 0.93 |
| 49 | miR-192 | 39,411.33 | 36,339.67 | 0.92 |
| 50 | miR-460 | 69.92 | 64.33 | 0.92 |
| 51 | miR-210 | 69.01 | 63.70 | 0.92 |
| 52 | miR-139 | 68.55 | 62.45 | 0.91 |
| 53 | miR-181 | 3269.19 | 2941.16 | 0.90 |
| 54 | miR-130 | 80.23 | 72.47 | 0.90 |
| 55 | miR-10 | 47,488.76 | 42,108.58 | 0.89 |
| 56 | miR-22 | 213,261.41 | 183,209.87 | 0.86 |
| 57 | miR-137 | 7.74 | 6.68 | 0.86 |
| 58 | miR-27 | 446.34 | 378.66 | 0.85 |
| 59 | miR-142 | 248.72 | 208.23 | 0.84 |
| 60 | miR-363 | 1285.32 | 1062.47 | 0.83 |
| 61 | miR-194 | 4759.55 | 3859.72 | 0.81 |
| 62 | miR-8 | 6261.60 | 5023.70 | 0.80 |
| 63 | miR-459 | 1.82 | 1.46 | 0.80 |
| 64 | miR-193a | 3.19 | 2.51 | 0.79 |
| 65 | miR-214 | 74.92 | 57.44 | 0.77 |
| 66 | miR-144 | 323.19 | 245.20 | 0.76 |
| 67 | miR-24 | 296.50 | 225.36 | 0.76 |
| 68 | miR-122 | 155,953.41 | 116,720.50 | 0.75 |
| 69 | miR-34 | 46.26 | 34.25 | 0.74 |
| 70 | miR-101 | 18,445.98 | 13,442.82 | 0.73 |
| 71 | miR-128 | 1516.45 | 1105.08 | 0.73 |
| 72 | miR-143 | 21,731.70 | 15,366.21 | 0.71 |
| 73 | miR-338 | 60.51 | 43.03 | 0.71 |
| 74 | miR-551 | 3.94 | 2.72 | 0.69 |
| 75 | miR-726 | 5.46 | 3.34 | 0.61 |
| 76 | miR-489 | 3.94 | 2.30 | 0.58 |
| **77** | **miR-184** | **112.68** | **45.53** | **0.40 *** |
| **78** | **miR-124** | **1.82** | **0.42** | **0.23 *** |

$$\text{TPM}=\frac{\text{Readcount X 1,000,000}}{\text{MappedReads}}$$

**Table S5.** Primers used for miRNA detection in this study.

| miRNA | ID | Primers (5′-3′) | Fold Changes by Deep Sequences |
|---|---|---|---|
| 1 | ccr-miR-365 | CGGTAATGCCCCTAAAAATCCTTAT | +2.57 |
| 2 | ccr-miR-196b | CGGTAGGTAGTTTCAAGTTGTTGGG | +2.75 |
| 3 | ID_LG1_121431 | CGGTTAATGGCAACTGTCAACTGTT | +13.77 |
| 4 | ID_LG9_186104 | CGGTGGAACCACTGACTTACATAG | +3.37 |
| 5 | ccr-miR-184 | TGGACGGAGAACTGATAAGGGC | −2.47 |
| 6 | ID_LG30_147265 | GCGGTTCCTATGCATATACCTCTTT | −4.18 |
| 7 | ID_LG27_175722 | CGGAAGTCAGTGGGAACTGAAACT | −5.57 |
| U6 RNA | U6 Forward | CGCTTCGGCAGCACATATAC | - |
| | U6 Reverse | TTCACGAATTTGCGTGTCA | - |
